# Novel *PRKAG2* variant presenting as liver cirrhosis: report of a family with 2 cases and review of literature

**DOI:** 10.1186/s12920-021-00879-1

**Published:** 2021-01-28

**Authors:** Zahra Beyzaei, Fatih Ezgu, Bita Geramizadeh, Alireza Alborzi, Alireza Shojazadeh

**Affiliations:** 1 Shiraz Transplant Research Center (STRC), Shiraz University of Medical Sciences, Shiraz, Iran; 2 Department of Pediatric Metabolism and Genetic, Faculty of Medicine, Gazi University, Ankara, Turkey; 3 Department of Pathology, Shiraz University of Medical Sciences, Khalili St., Research Tower, Seventh Floor, Shiraz Transplant Research Center (STRC), Shiraz, Iran; 4 Student Research Committee, Shiraz University of Medical Sciences, Shiraz, Iran

**Keywords:** *PRKAG2* syndrome, Cirrhosis, Wolff–Parkinson–White syndrome, Targeted gene sequencing

## Abstract

**Background:**

Mutations in the PRKAG2 gene encoding the 5′ Adenosine Monophosphate-Activated Protein Kinase (AMPK), specifically in its γ2 regulatory subunit, lead to Glycogen storage disease of heart, fetal congenital disorder (PRKAG2 syndrome). These mutations are rare, and their functional roles have not been fully elucidated. PRKAG2 syndrome is autosomal dominant disorder inherited with full penetrance. It is characterized by the accumulation of glycogen in the heart tissue, which is clinically manifested as hypertrophic cardiomyopathy. There is little knowledge about the characteristics of this disease. This study reports a genetic defect in an Iranian family with liver problems using targeted-gene sequencing.

**Case presentation:**

A 4-year-old girl presented with short stature, hepatomegaly, and liver cirrhosis. As there was no specific diagnosis made based on the laboratory data and liver biopsy results, targeted-gene sequencing (TGS) was performed to detect the molecular basis of the disease. It was confirmed that this patient carried a novel heterozygous variant in the *PRKAG2* gene. The echocardiography was a normal.

**Conclusion:**

A novel heterozygous variant c.592A > T (p.Met198Leu) expands the mutational spectrum of the *PRKAG2* gene in this family. Also, liver damage in patients with PRKAG2 syndrome has never been reported, which deserves further discussion.

## Background

Glycogen storage disease of heart, lethal congenital (PRKAG2 syndrome) is a disorder of glycogen metabolism, essentially heart-specific [1]. It has autosomal dominant inheritance with complete penetrance [2]. The disease is caused by mutations in the *PRKAG2* gene, which encodes the non-catalytic subunit γ2 activated by the protein kinase AMP. The gene of *PRKAG2* has 22 exons located in the 7q36.1 region. The prevalence of this disorder is rare, which is reported as 0.23–1% in patients with fatal infantile cardiomyopathy [3]. To the best of our knowledge, less than 200 patients with genetically confirmed PRKAG2 syndrome have been reported so far [4]. Clinical presentation of patients often represents during late adolescence and rare manifestations during childhood were described [5–7]. Mutation in the *PRKAG2* gene is identified mainly by cardiac symptoms. To date, other symptoms such as skeletal muscle involvement, and sometimes-enlarged kidneys were reported; however, the exact spectrum of signs are not fully elucidated yet [2, 3]. In rare, sporadic cases, heart failure and respiratory compromise have been reported, which leads to death within a few weeks or months after birth [8–10].

In this study, we investigated the affected case in an Iranian family with a novel heterozygous *PRKAG2* mutation who presented with liver problems.

## Case presentation

A 4-year-old Iranian girl was referred to our center for genetic analysis presented with a history of mild hepatomegaly and short stature. She is the second child of a non-consanguineous family. She was delivered following a normal vaginal delivery after term pregnancy with a birth weight of 2.50 kg, and height of 46 cm. No hypoglycemia was noted in the perinatal period and the postnatal transition.

At the age of one, she was admitted because of hepatomegaly, diarrhea, and developmental delay. Biochemical tests revealed alanine aminotransferase (ALT) 13 IU/L, and aspartate aminotransferase (AST) 63 IU/L. Triglyceride was normal (66 mg/dl) with a normal cholesterol level (136 mg/dl), but a high level of LDH (1024 U/L). Peripheral blood smear showed hypochromic microcytic anemia (hemoglobin of 10.8 g/dl and a hematocrit of 34.6%, the mean corpuscular volume of 60.40 fl, and low mean corpuscular hemoglobin 18.81 pg) and normal platelet count (221,000/µL). Hence, blood gas analysis and electrolyte levels were normal, but a low level of CPK (18 U/L), was documented as shown in Table 1.

**Table 1:** Lab test results of the affected member in the current family with PRKAG2 syndrome

Analysis	Result	Reference range
Alanine aminotransferase	13 IU/L	Up to 31 IU/L
Aspartate aminotransferase	63 IU/L	Up to 31 IU/L
Triglyceride	66 mg/dl	35–135 mg/dl
Cholesterol	136 mg/dl	130–200 mg/dl
LDH	1024 U/L	Up to 850 U/L
CpK	18 U/L	Female: 24–195 U/L
Hemoglobin	10.8 g/dl	12.1–15.1 g/dl
Hematocrit	34.6%	35.5–44.9%
Mean corpuscular volume	60.04 fl	80–96 fl
Low mean corpuscular hemoglobin	18.81 pg	27–33 pg
Platelet count	221,000/µL	150,000–450,000/µL

Urine amino acid analysis by chromatography presented a weak band in cysteine and moderate bond in arginine. Lung fluid showed inflammatory cells mostly PMNs admixed with respiratory columnar cells and squamous cells in the proteinaceous background. No fungal elements were identified. Histology of the liver tissue revealed cirrhosis. According to clinical presentation and liver biopsy, storage disease was suggested by the local pediatrician without genetic analysis, so treatment with frequent feeds and cornstarch was initiated.

At the age of 3, Targeted gene sequencing (TGS) with a custom-targeted Ion AmpliSeq™ panel was performed. The panel included 7219 amplicons covering 450 genes associated with Inborn Metabolic Diseases consisting of glycogen storage disorders genes with hepatic involvement. Sanger sequencing validated identified the variants, using an ABI Prism 3500 Genetic Analyzer (Applied Biosystems, Foster City, CA, USA). Analyses were done using an Ion Torrent 540 chip (Life Technologies, Guilford, CT, South San Francisco, CA). The human GRCh37/hg 19 was used as the reference. Polyphen2, SIFT, and Mutation Taster were used for in silico analysis, GERP and Phastcons scores were used to evaluate the conservation of the variants. The population frequency of each variation was evaluated, using data from the gnomAD database. ACMG guidelines were used for variant interpretation [11]. The sequence variants were described according to the Human Genome Variation Society Nomenclature [12]. The sequence variant RefSeq NM_001040633.1 was used. Interestingly, a novel heterozygous variant c.592A > T (p.Met198Leu) was detected, in the exon 1 of the *PRKAG2* gene, by TGS and suggested PRKAG2 syndrome*.* Further functional tests are needed.

On admission, the patient’s echocardiogram revealed normal heart function with very mild MR, and TR. Tandem mass spectrometry (MS/MS) analysis using urine revealed normal results for Glc4.

**Fig. 1:**
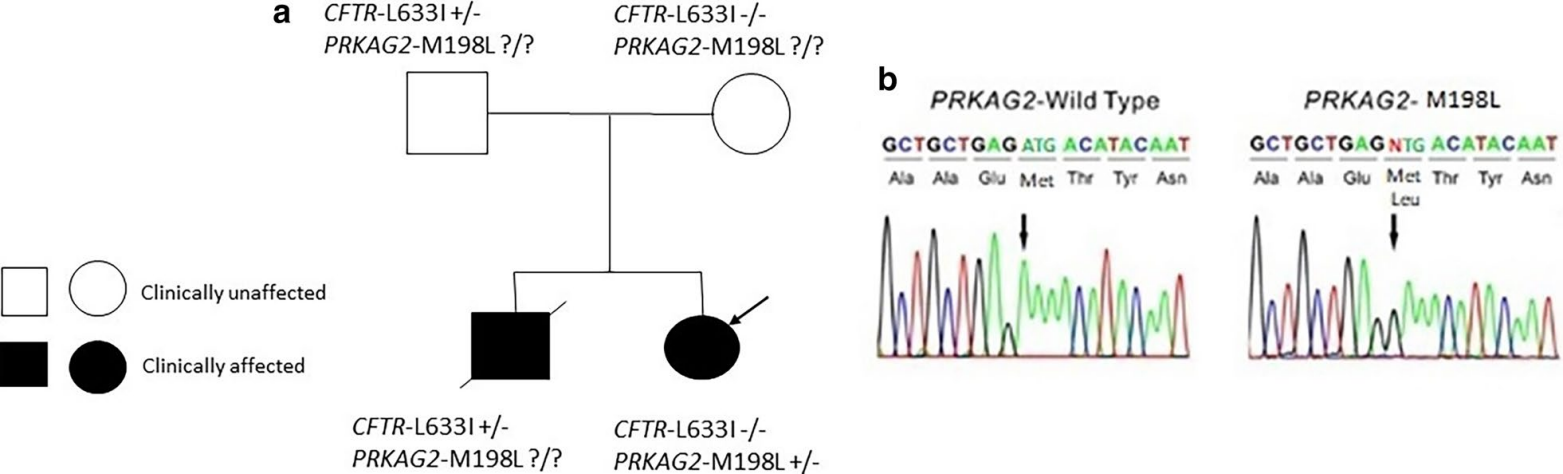
Genetic analysis identified a novel de novo *PRKAG2* mutation. **a** Family pedigree of the *PRKAG2* mutant carrier. Black arrow shows proband and slants denote dead individuals. **b** DNA chromatogram shows a heterozygous A-to-T transition at nucleotide 592 of *PRKAG2*, predicting a substitution of a methionine for isoleucine at residue 198 (p.Met198Lue) of the AMP-activated protein kinase (AMPK) γ-2 subunit (M198L). + indicates mutation positive, − indicates mutation negative, and ? indicates no analysis. PRKAG2, RefSeq NM_001040633.1

Past medical family history of the patient revealed that proband had a sibling (boy) who expired at the age of 15 years old with pulmonary and liver symptoms. Figure 1 presents the pedigree of the family with 2 affected children. The proband's brother had a heterozygous variant c.1897C > A (p.Leu633Ile) which was detected in the exon 14 of the *CFTR* gene by WES. Their parents were also examined for this mutation. The father of the family carried a heterozygous mutation too, but he was always healthy with no pulmonary symptoms. The proband's brother also had a liver biopsy, which revealed cirrhosis with no known underlying cause. He had undergone liver transplantation at the age of 6. At the age of 14, his echocardiogram showed mild MR, TR, and severe pulmonary hypertension. Finally, the proband's brother died at age 15 before mutation analysis for the *PRKAG2* gene. Proband was normal for mutation on the *CFTR* gene by WES (Fig. 1).

## Discussion and conclusions

The present study of an Iranian family identifies, for the first time, a novel missense variant of the *PRKAG2* gene (c.592A > T, p.Met198Leu), which was identified by TGS. It has been reported to cause glycogen storage disease of the heart, fetal congenital disorder (PRKAG2 syndrome), and atypical involvement of the liver.

The γ2-regulatory subunit of AMP-activated protein kinase (AMPK) is an enzyme that regulated the amount of ATP, which is essential for metabolic activity. It is encoded by the *PRKAG2* gene [5]. Mutations in this protein kinase are related to a wide variety of manifestations, including glycogen accumulation, Wolff–Parkinson–White syndrome, and conduction disorder [2, 3, 5]. However, conduction disorder is not always available in PRKAG2 syndrome, and it may appear in the future [13, 14]. Electrocardiographic abnormalities are usually in PRKAG2 syndrome, but we did not find it in our proband. According to reports, the mechanism of impaired glucose metabolism and increased glycogen storage in cells caused by *PRKAG2* mutations is still unclear [15].

To date, about 21 mutations have been demonstrated to be associated with PRKAG2 syndrome [16]. Most of these mutations are missenses, and the most abundant ones are p.R302Q [16] and p.N488I [17]. Hence, the clinical manifestations were different in the families carrying the mutations in the *PRKAG2* gene. Even in the same family, the affected patients did not present the same manifestations with similar mutations. Cardiac conduction disorder is the main feature of affected members, as reported previously. To find and carefully evaluate all reported mutations and unusual effects on the presentation of *PRKG2*, we did a literature search until October 2020. Sporadic cases were reported to have enlarged and dysmorphic kidneys and chronic kidney disorder, as presented in Table 2 [2, 4]. In this study, we presented a family with two children both having liver cirrhosis and pulmonary symptoms with no cardiac problem. Further research and more cases are needed to determine the clinical correlation between the presence of a detected novel mutation and the adverse outcomes of a patient with PRKAG2 syndrome. The integration of biochemical, transcriptional, and functional datasets using human induced pluripotent stem cells (iPScs), micro-tissues, and mouse models allowed us to analyze how mutations produce the phenotypes observed in PRKAG2 mutations.

**Table 2:** Unusual features of patients with PRKAG2 syndrome

Report	Country/year	PRKAG2 mutation/SNP	Unusual event
Burwinkel et al. [2]	US/2005	R531Q	Enlarged and dysmorphic kidneys, Pulmonary edema
Köttgen et al. [4]	US/2010	SNP rs7805747	Chronic kidney disease (CKD)
Current study	Iran/2020	Met198Leu	Liver cirrhosis

Our case shows that molecular analysis (especially using TGS) is an important method to diagnose GSD subtypes. Early genetic diagnosis of TGS has many benefits, including time- and cost-effectiveness, correct treatment, accurate recurrence risk recommendations, and screening of the patients where appropriate [18]. Unfortunately, we do not have the genetic analysis of parents and expired brother to determine whether the expired brother had the same mutation or not.

In conclusion, we are reporting a novel variant of *PRKAG2* that is associated with *PRKAG2* syndrome in an Iranian family. Molecular screening for *PRKAG2* mutations may be considered in patients who have liver problems. The case highlights the advantage of targeted sequencing in diagnosing a patient with PRKAG2 syndrome, which may present unusual manifestation.

## Data Availability

The data that support the findings of this study are available from Gazi University of Medical Sciences but restrictions apply to the availability of these data, which were used under license for the current study, and so are not publicly available. Data are however available from the authors upon reasonable request and with permission of Gazi University of Medical Sciences. The datasets generated and/or analyzed during the current study are available in the Genbank repository (GRCh37/hg19, https://www.ncbi.nlm.nih.gov/genome/guide/human/) for PRKAG2: NM_001040633.1 and CFTR: NM_ 000492.4 (https://www.ncbi.nlm.nih.gov/nuccore).

## Abbreviations

AMPK: AMP-activated protein kinase
H&E: Hematoxylin and Eosin
HCM: Hypertrophic cardiomyopathy
iPScs: Induced pluripotent stem cells
LAD: Left anterior descending artery
LVH: Left ventricular hypertrophy
PAS: Periodic acid-Schiff
PFIC: Progressive familial intrahepatic cholestasis
PMNs: Polymorphonuclear
PRKAG2: Protein kinase AMP-activated non-catalytic subunit gamma 2
WPW: Wolff–Parkinson–White

## References

[1] Sri A, Daubeney P, Prasad S, Baksi J, Kinali M, Voges I (2019). A case series on cardiac and skeletal involvement in two families with PRKAG2 mutations. Case Rep Pediatr.

[2] Burwinkel B, Scott JW, Buhrer C, van Landeghem FK, Cox GF, Wilson CJ, Grahame HD, Kilimann MW (2005). Fatal congenital heart glycogenosis caused by a recurrent activating R531Q mutation in the gamma 2-subunit of AMP-activated protein kinase (PRKAG2), not by phosphorylase kinase deficiency. Am J Hum Genet.

[3] Murphy RT, Mogensen J, McGarry K (2005). Adenosine monophosphate-activated protein kinase disease mimicks hypertrophic cardiomyopathy and Wolff–Parkinson–White syndrome. J Am Coll CardioL.

[4] Kottgen A, Pattaro C, Bo¨ger CA, Fuchsberger C. New loci associated with kidney function and chronic kidney disease. Nat Genet. 2010;42:376–384. 10.1038/ng.568 PMC2997674 20383146

[5] Porto AG, Brun F, Severini GM, Losurdo P, Fabris E, Taylor M, Mestroni L, Sinagra G (2016). Clinical spectrum of PRKAG2 syndrome. Circ Arrhythm Electrophysiol.

[6] Beyzaei Z, Geramizadeh B (2019). Molecular diagnosis of glycogen storage disease type I: a review. EXCLI J.

[7] Pöyhönen P, Hiippala A, Ollila L (2015). Cardiovascular magnetic resonance findings in patients with PRKAG2 gene mutations. J Cardiovasc Magn Reson.

[8] Mizuta K, Hashimoto E, Tsutou A, Eishi Y, Takemura T, Narisawa K, Yamamura H (1984). A new type of glycogen storage disease caused by deficiency of cardiac phosphorylase kinase. Biochem Biophys Res Commun.

[9] Bührer C, van Landeghem FKH, Felderhoff-Mueser U, Stadelmann C, Obladen M (2003). Fetal bradycardia at 28 weeks of gestation associated with cardiac glycogen phosphorylase b kinase deficiency. Acta Paediatr.

[10] Elleder M, Shin YS, Zuntova A, Vojtovic P, Chalupecki V (1993). Fatal infantile hypertrophic cardiomyopathy secondary to deficiency of heart specific phosphorylase b kinase. Virchows Archiv A Pathol Anat.

[11] Richards S, Aziz N, Bale S, Bick D (2015). Standards and guidelines for the interpretation of sequence variants: a joint consensus recommendation of the American College of Medical Genetics and Genomics and the Association for Molecular Pathology. Genet Med.

[12] Den Dunnen JT, Antonarakis SE (2000). Mutation nomenclature extensions and suggestions to describe complex mutations: a discussion. Hum Mut.

[13] Mehdirad AA, Fatkin D, DiMarco JP, MacRae CA, Wase A, Seidman JG, Seidman CE, Benson DW (1999). Electrophysiologic characteristics of accessory atrioventricular connections in an inherited form of Wolff–Parkinson–White syndrome. J Cardiovasc Electrophysiol.

[14] Zhang LP, Hui B, Gao BR (2011). High risk of sudden death associated with a PRKAG2-related familial Wolff–Parkinson–White syndrome. J Electrocardiol.

[15] Ha ACT, Renaud JM, Dekemp RA, Thorn S, Dasilva J, Garrard L (2009). In vivo assessment of myocardial glucose uptake by positron emission tomography in adults with the PRKAG2 cardiac syndrome. Circ Cardiovasc Imaging.

[16] Pöyhönen P, Hiippala A, Ollila L, Kaasalainen T, Hänninen H, Heliö T, Tallila J (2015). Cardiovascular magnetic resonance findings in patients with PRKAG2 gene mutations. J Cardiovasc Magn R.

[17] Arad M, Moskowitz IP, Patel VV, Ahmad F, Perez-Atayde AR, Sawyer DB, Walter M, Li GH, Burgon PG, Maguire CT (2003). Transgenic mice overexpressing mutant PRKAG2 define the cause of Wolff–Parkinson–White syndrome in glycogen storage cardiomyopathy. Circulation.

[18] Beyzaei Z, Geramizadeh B, Karimzadeh S (2020). Diagnosis of hepatic Glycogen storage disease patients with overlapping clinical symptoms by massively parallel sequencing: a systematic review of literature. Orphanet J Rare Dis.

